# *In-vitro* digestion models: a critical review for human and fish and a protocol for *in-vitro* digestion in fish

**DOI:** 10.1080/21655979.2021.1940769

**Published:** 2021-06-30

**Authors:** Ricky Wang, Mahtab Mohammadi, Amir Mahboubi, Mohammad J. Taherzadeh

**Affiliations:** Swedish Centre for Resource Recovery, University of Borås, Borås. Sweden

**Keywords:** Bioreactors, *in-vitro* digestion, gastrointestinal model, fish meal replacement, protein digestibility

## Abstract

Digestive systems in human, animals, and fish are biological reactors and membranes to digest food and extract nutrients. Therefore, static and dynamic models of *in-vitro* digestion systems are developed to study e.g. novel food and feed before *in-vivo* studies. Such models are well developed for human, but not to the same extent for animals and fish. On the other hand, recent advances in aquaculture nutrition have created several potential fish meal replacements, and the assessment of their nutrient digestibility is critical in the application as a fish meal replacement. Using an *in-vitro* method, the assessment of an ingredient digestibility could be faster and less expensive compared to using an *in-vivo* experiment. An *in-vitro* method has been widely used to assess food nutrient digestibility for humans; however, its application for fish is still in the early stages. Both the human and fish as monogastric vertebrates share similar gastrointestinal systems; thus, the concept from the application for humans could be applied for fish. This review aims to improve the *in-vitro* digestion protocol for fish by adapting the concept from then study for humans, summarizing the current available *in-vitro* digestion model developed for human and fish *in-vitro* digestion study, identifying challenges specifically for fish required to be tackled and suggesting an engineering approach to adapt the human *in-vitro* gastrointestinal model to fish. Protocols to conduct *in-vitro* digestion study for fish are then proposed.

## Graphical abstract

**Figure:**
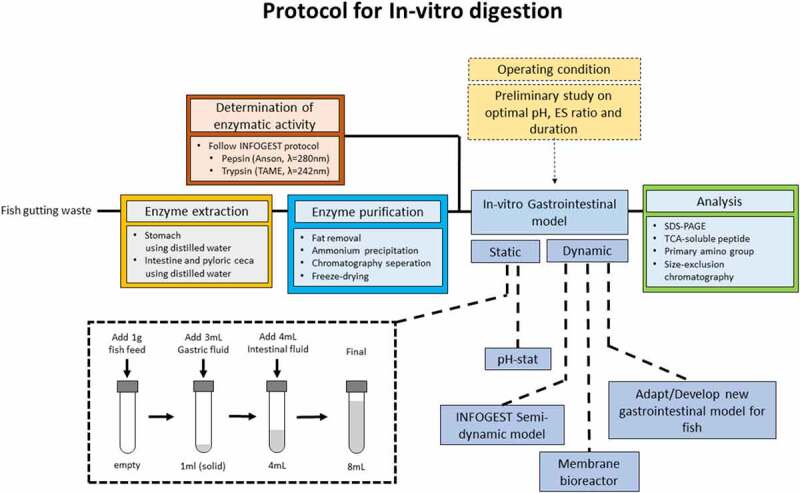

## 1. Introduction

Two of the global Sustainable Development Goals (SDG) are to achieve food security and improved nutrition (SDG 2) and conserving marine resources (SDG 14). Sustainable fish aquaculture is one of the action to achieve both goals by substituting wild-caught fish, which has been regulated to prevent overfishing, illegal, unreported and unregulated fishing, and destructive fishing practices [1]. In fact, the aquaculture industry is one of the fastest-growing industries, projected to grow by one-third in a decade by 2030 [2]. The main challenges of fish aquaculture are providing the protein for the fish feed, which is currently provisioned from fish meal supplied from wild-caught fish, thus considered as unsustainable [3]. Moreover, the limited supply of fish meal to satisfy the aquaculture growth resulted in a higher price of fish feed [4]. Therefore, replacing the demands for fish meal is the challenge in aquaculture expansion to achieve global sustainability goals, including the conservation of using marine resources [5].

Several sustainable alternatives for either partial or total replacement of protein from fish meal has been proposed in the literature. Animal-based protein, mostly the by-products of the food industry such as poultry meal [6], meat and bone meal [7–9], and feather meal [10], are reported to be suitable as a fish meal replacement. However, those protein sources are costly and have limited availability [11]. Plant-based protein such as soybean, lupine, rapeseed, canola, pea, corn gluten, wheat gluten, and cottonseed has received significant attention as a fish meal replacement because of their availability, low price, and high-protein content [3]. However, the plant-based protein was reported to have low digestibility and contain antinutrients, limiting their application as a fish meal replacement [3]. Recent advances have proposed the usage of sustainable protein sources, such as insect meal [12], single-cell proteins made of bacteria, yeast, microalgae [13], and edible fungi [11,14]. Based on the nutritional profile – amino acid and fatty acid profile, prebiotic and immunomodulant properties, these sustainable novel ingredients have been considered as high-quality fish meal replacements. However, to conclude their suitability as a fish meal replacement, those ingredients’ digestibility must be thoroughly studied for the fish species.

The nutrient digestibility assessment is typically conducted *in-vivo* by feeding trials on the fish species of interest, measuring the growth rate, fecal content, and fish survival rate [15]. Even though it provides a more accurate and representative result, the *in-vivo* method is lengthy and expensive [16]. The alternative is the *in-vitro* method, conducted by simulating the physiology of fish gastrointestinal tract using laboratory equipment. This method has gained widespread attention to evaluate a potential food or feed product’s digestibility for humans [17], terrestrial animals such as poultry [18], ruminants [19], and aquatic species [20], including fish, prawns, and mollusks. The main drawback of an *in-vitro* method is that it could not reflect the full complexity of the digestion process that occurs *in-vivo*, thus lacking the accuracy and reliability compared to an *in-vivo* method.

Nevertheless, due to its low cost, no ethical restriction, and relatively simple execution, the *in-vitro* digestibility test is suitable for preliminary studies, which require a considerable amount of samples to be evaluated [17]. It also allows a controlled experiment to study the mechanism of hydrolysis of protein, lipid, and carbohydrate in food or feeds products [20]. The necessity of understanding the fates of the ingested product in humans has given rise to several attempts to develop an *in-vitro* model for the human gastrointestinal system in the past few decades [21]. It has been widely applied to simulate the digestion process in humans, particularly in food science, nutrition, toxicology, pharmacology, and microbiology [22].

**Figure 1.**
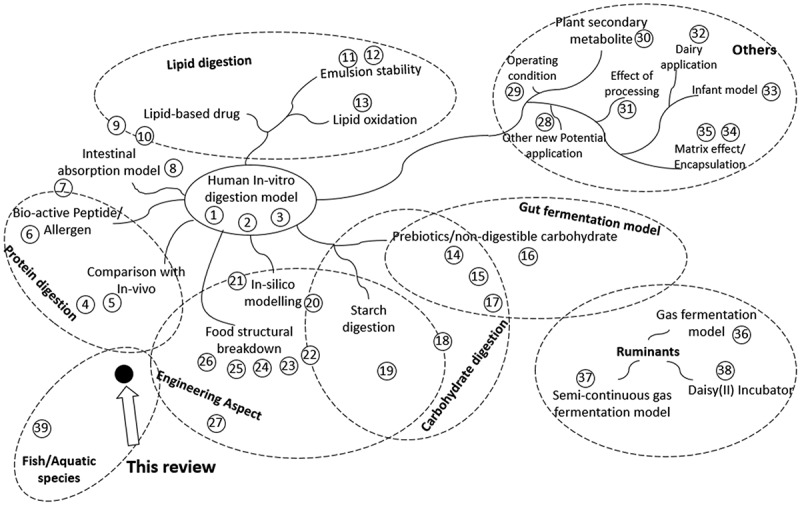
Current review articles on *in-vitro* gastrointestinal digestion of humans, ruminants, and aquatic species for several different topics. Proximity of the references to different topics indicates the salient feature of the review articles encompassing multiple topics. Numbered references are listed in Supplementary References

Similar interest has also been raised in the aquaculture industry to utilize an *in-vitro* method to assess the fish feed digestibility [20]. However, based on the sheer number of publications, the study on *in-vitro* digestion for fish is still in its infancy compared to humans. The *in-vitro* digestion studies for human has been applied throughout several different applications (Figure 1). Moreover, they were well documented with several globally practiced protocols, while the protocols for fish was limited and highly varied between different authors [23]. Both fish and humans are monogastric vertebrates, sharing a similar gastrointestinal tract with differences in the anatomical, physiological, and enzymatic features. Given the huge knowledge gap between human and fish, the *in-vitro* digestion method for fish could be improved by applying some of the concepts from the study for humans.

This review aims to review the current practice of *in-vitro* digestion study on both humans and fish, exploring potential options for applying *in-vitro* digestion methodology developed for humans on fish. Firstly, the current developed *in-vitro* gastrointestinal model for humans is elaborated. The recent studies on *in-vitro* fish digestion are then reviewed, followed by pointing out several current challenges in conducting an *in-vitro* digestion study on fish. An engineering approach of *in-vitro* digestion is then elaborated as the tool to adapt the developed human gastrointestinal model on fish. Lastly, a protocol for the *in-vitro* digestion for fish is suggested based on the current practice on humans.

## 2. *In-vitro* Gastrointestinal Model

The gastrointestinal tract of a monogastric animal, including humans and fish, consists of a mouth, an esophagus, a stomach, and intestines. Food entering the mouth is masticated and physically broken down. Saliva is secreted to hydrolyze the starch present in the food, forming a bolus that is transferred to the stomach through the esophagus. In the stomach, the bolus is mixed with the acidic gastric juice; together with gastric enzyme, mainly pepsin, to digest the protein. After that, chyme – the product of the gastric phase – enters the first small intestine section called the duodenum and has the pH neutralized to 7 prior to mixing with bile and pancreatic enzymes, which include trypsin, chymotrypsin, amylase, lipase, phospholipase, and several other proteases. Along the later sections of the small intestine, namely jejunum and ileum, nutrients continue being enzymatically hydrolyzed while absorbed by the intestinal absorptive cells. The undigested material is then continued to large intestines, where it is anaerobically fermented by the microorganism population, producing short-chain fatty acids and vitamins, which can be absorbed by the intestinal cell. The leftover material is then excreted as feces.

An *in-vitro* gastrointestinal model mimics the complex process of food digestion that occurs along the gastrointestinal tract. Based on the complexity, the *in-vitro* digestion model can be categorized into static and dynamic digestion models.

### 2.1. Static Digestion Model

The static digestion model is the simplest model to simulate the digestion process. Food is first added into a reaction vessel (beaker, Erlenmeyer flask, or test tube). Then, the digestive fluid and enzymes are added for each digestion phase (oral, gastric, and intestinal). The pH could be either left uncontrolled or kept constant using a pH-stat system. Briefly, as an example, 1 g of the sample is added into a test tube and mixed with 1 mL of simulated salivary fluid at pH 7 for 2 min at 37°C. Two milliliters of simulated gastric fluid and pepsin are then added to the same test tube, and the pH is adjusted to 3.0 with HCl, reaching the total volume of 4 ml for the gastric phase with pepsin activity of 2000 U/mL. After incubation for 120 min, the pH is adjusted back to 7 with NaOH, then 4 mL of simulated intestinal fluid, which contains the pancreatin and bile salts, are then added to simulate the intestinal phase and incubated for 120 min. The final volume of the intestinal phase is 8 mL with trypsin activity of 100 U/mL [24].

One of the static digestion model challenges is determining the experimental condition (pH, amount of enzymes, duration). It needs to resemble the *in-vivo* digestion physiology present in humans or fish. Previously, there was a lack of a standardized protocol; each author adopted a different protocol with several slight but critical variations [24,25], causing results from different studies impossible to be compared. In response to that, an international network INFOGEST was established, consisting of experts from multidisciplinary fields from 32 countries. One of the primary outcomes was an international consensus to harmonize the *in-vitro* digestion protocol simulating an adult human digestion process, known as INFOGEST methods [24,26]. There are also other standardized methods such as United States Pharmacopoeia methods and Unified BARGE Method. However, those methods are not suitable to assess food products as they were developed for different purposes: pharmaceutical products and contaminants in a soil sample or mycotoxins in food [24].

Due to its simplicity, the static digestion model is suitable for *in-vitro* digestion studies with the research objective to screen, compare, or build a hypothesis where a considerable number of samples need to be analyzed. In particular, static digestion model is widely used to evaluate the effect of food processing on the nutrient bioaccessibility (nutrient released from the food matrix), bioavailability (including nutrient absorption), or allergenic peptides. Several food processing techniques, such as heat-treatment [27–30], drying [31], curing/aging [32], ultrasonication [33,34], and protein extraction [35], have been employed for numerous food products.

Despite its advantage as a simple and fast protocol, static digestion method cannot mimic the complex digestion processes present in the *in-vivo* condition [24]. It assumes that between the pH changes instantaneously between different digestion phases, lacking the gradual addition of gastric fluid (acid and pepsin) and gastric emptying. The intestinal phase does not include continuous nutrient removal, resembling the absorption process by intestinal cells. These shortcomings render the method unsuitable for detailed analysis of the different digestion process stages [36].

### 2.2. Dynamic Digestion Model

The dynamic digestion model is a computer-controlled model capable of simulating the complex digestion processes, which are not included in the static model, such as gastric mixing, gradual secretion of gastric juice, gastric emptying, and nutrient absorption. The significance of those phenomena will be discussed in the next section. Operating in a dynamic digestion model indubitably has better accuracy, represents the *in-vivo* conditions, and provides digestion kinetics compared to the static digestion model. However, it is time-consuming, highly complex, and requires more expensive enzymes, thus far less accessible than a static model [36]. Dynamic gastrointestinal model has been used to investigate the process of digestion in details, such as properties of lipid emulsion for lipid-soluble nutrients [37], novel food or drug encapsulation technique [38,39], kinetic of structural changes and release of proteins and lipids [40], kinetics of lipid oxidation during digestion process [41], possible interactions between different food on nutrients digestibility [42], and effect of food rheological properties [43–45] .

Several dynamic digestion models have been proposed for the *in-vitro* digestion study on humans. They are categorized as mono-compartmental and multi-compartmental models [21]. Most of the mono-compartmental models simulate gastric digestion in detail, including the physical gastric contraction, the fluid mechanics of mixing, the gradual addition of gastric juice, and gastric emptying. Several gastric models have been proposed (Table 1). All of the gastric models had the main chamber made up of an elastic material and incorporated with the gradual addition of gastric juice and pH control systems. HGS has a significantly higher gastric volume of 7 Liters, capable of simulating an actual human full meal, compared to other gastric models with chamber volume ranging from 500 to 900 ml. Each of the developed gastric models has different apparatus to simulate the gastric contraction, such as water pressure (1), rollers (2 4), pistons (3 5), and ropes (6). Some models resemble the J-shaped anatomical feature of the human stomach made by 3D printing (3 4 5 6). There are also several options to simulate the small opening pyloric valve and gastric emptying, for instance, using an elastic annulus (1 6), mesh filter (2), or a tapered structural design (3 4).

**Table 1.** Dynamic *in-vitro* gastrointestinal model

Code*		Name	Reference
1	Mono-compartmental	Dynamic Gastric Model (DGM)	[153]
2		Human Gastric Simulator (HGS) v1.0 and v.2.0	[51,154]
3		Gastric Simulation Model (GSM)	[155]
4		Gastric Digestion Simulator (GDS)	[156]
5		*In-vitro* Mechanical Gastric System (IMGS)	[157]
6		Dynamic *In-vitro* Human Stomach (DIVHS)	[158,159]
7		Artificial Colon (ARCOL)	[160]
8		Dynamic Colon Model (DCM)	[161]
9	Multi-compartmental	Dynamic gastrointestinal digester (DIDGI®)	[59]
10		TNO gastrointestinal model (TIM-1, TIM-2, tiny-TIM)	[60,162]
11		Simulator of the Human Intestinal Microbial Ecosystem (SHIME)	[163]
12		Simulator of the Gastrointestinal Tract (SIMGI)	[164]
13		Engineered Stomach And Small Intestine Model (ESIN)	[165]
14		SimuGIT	[39]
15		Membrane bioreactor with dialysis cell	[102,103]
*The references in parentheses throughout features of dynamic digestion models indicate the code number in this table			

Another organ that is widely modeled for an *in-vitro* study is the colon. Several models were proposed, including TIM-2, artificial colon (ARCOL), Dynamic Colon Model (DCM), and several others [46–49]. ARCOL simulates the colon fermentation in a bioreactor equipped with hollow fiber membranes using fresh feces from volunteers as the inoculum. DCM is a biomechanical model made up of 10 membrane segments inflated by computer-controlled hydraulic syringes, simulating the peristaltic movement validated with Magnetic Resonance Imaging (MRI).

In a multi-compartmental model, the digestion process is simulated in several reaction chambers representing the mouth, stomach, and intestines. Several multi-compartmental gastrointestinal models have been developed (Table 1). Each model is unique in terms of the reaction vessel, contraction and mixing mechanism, gastric emptying, the inclusion of oral phase, nutrient absorption, or large intestine fermentation. The reaction chamber can be modeled with several commercial computer-controlled stirred tank bioreactors (9 11 12 13 14 15) or custom design elastic chambers with water pressure modulation (10). The nutrient absorption process has been modeled in several configurations, such as an external hollow fiber membrane filtration (10), a membrane bioreactor unit (15), or a tubular bioreactor made of a ceramic microfiltration membrane (14). The colonic fermentation is included in several models (11 12).

Recently, a standardized semi-dynamic model for *in-vitro* digestion study on humans was proposed [36]. Semi-dynamic means that the model simulates the gastric phase dynamic process, but not the intestinal phase: the gradual addition of gastric juice was achieved using an automatic titrator, mixing was carried out by a 3D-printed stirrer, gastric emptying was performed by manual periodical transfer of the product from the gastric phase into several test tubes. Each test tube then proceeded with the same standardized static digestion protocol of the intestinal phase. This semi-dynamic model is easily replicated as it only requires standard chemical laboratory equipment.

### 2.3. Engineering Aspect of the Dynamic Model

Developing a dynamic *in-vitro* digestion model requires mimicking the physical and biochemical processes in the gastrointestinal tract. Validating an *in-vitro* model with the *in-vivo* experiment is commonly carried out by only comparing the biochemical data of the digesta. It is beneficial to also compare the physical properties of the digesta, for instance, digesta viscosity and particle size distribution. Moreover, these physical properties allow the quantification of the physical process in the dynamic model to coincide with the *in-vivo* condition.

**Figure 2.**
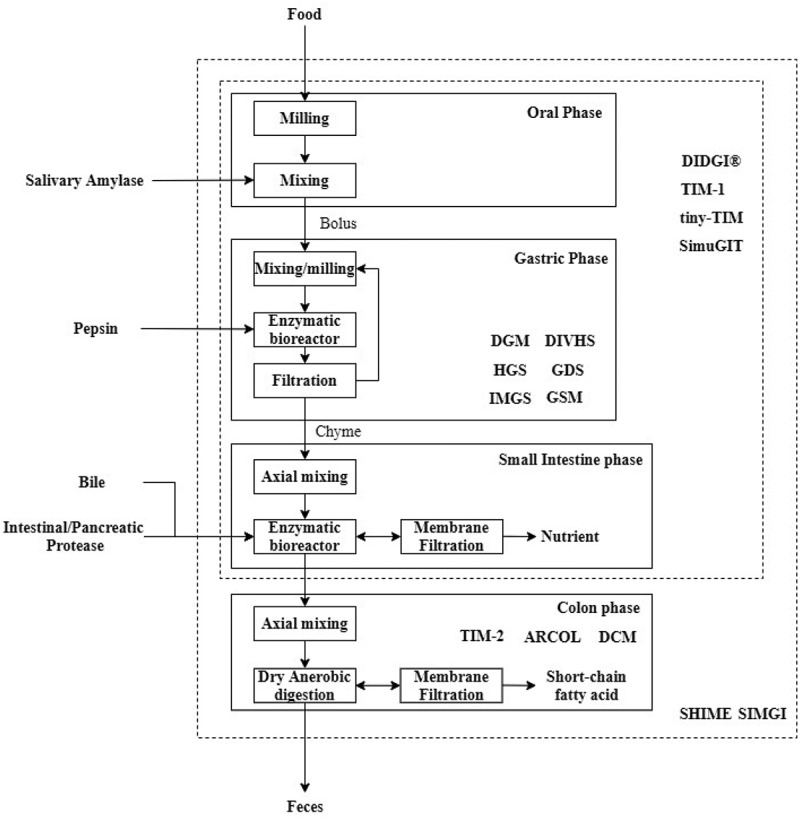
Block flow diagram of the human gastrointestinal digestion process modeled as various unit operations adapted from Bornhorst et al. [50], with the simulation scope of several current developed dynamic *in-vitro* gastrointestinal models

The complex digestion processes can be identified as several fundamental unit processes such as size reduction, mixing, transport, filtration, biochemical reaction, and microbial fermentation (Figure 2). These processes can be characterized by using an engineering approach widely used in food or chemical process industries. Bornhorst et al. [50] reviewed several engineering approaches to model the *in-vitro* digestion process.

The particle size distribution function is the key characterization parameter of the size-reduction process (e.g. in the cement and mining industry). The parameter could be adopted for the *in-vitro* digestion process to estimate the degree of food breakdown during the oral and gastric phases. The *in-vivo* data of this parameter allows the design of the *in-vitro* oral and gastric size-reduction process. During the *in-vitro* digestion process, the kinetics of particle size distribution could reveal the mechanism of food breakdown during the gastric phase [51].

Mixing is an important factor since it assists with the breakdown of the food matrix and promotes gastric enzyme diffusion [51,52]. Mixing is a common unit processing in nearly all industries. One of the parameters to quantify the degree of mixing is the mixing index. The mixing index can be calculated with several methods based on the application as reviewed by Bornhorst [53]. Gradual secretion of gastric juice simulates a gradual decrease in pH as the bolus enters the gastric compartment to final pH 3. This gradual pH decrease provides extra time for salivary amylase, gastric lipase to maintain their enzymatic activity to hydrolyze starch and lipids before they are inactivated by acidic pH. Gastric lipase contributes about 25% of total food lipid digestion for humans [24]. The parameter that quantifies the gradual decrease in pH is the buffering capacity. Buffering capacity of food is measured by acidic titration, with several calculation methods were proposed [54].

Gastric emptying simulates the gradual transfer of chyme from the gastric phase into the duodenum. It is known to significantly affect the accuracy of the *in-vitro* digestion models [24,55,56]. For example, in the case of milk protein digestion, gastric emptying causes a faster release of acid-soluble proteins (ex. whey protein) into the intestinal phase while prolonging the duration of acid-coagulated protein (ex. casein) in the stomach. This circumstance was validated in an *in-vivo* trial by blood plasma measurement [57]; thus, whey and casein are termed as fast and slow protein, respectively.

Gastric emptying has been studied in detail for several important goals, such as in the design for oral drug delivery and probiotics survival. Gastric emptying by the pyloric sphincter can be represented by the filtration unit with a specific mesh size (1–3 mm for humans). There are several mathematical models for gastric emptying, ranging from the simplest based on food caloric content [36] to a stochastic model [58]. The modified power-exponential model has been widely used in developing a dynamic *in-vitro* gastrointestinal model [59–61]. The gradual transfer of the gastric digesta into the duodenum causes the intestinal enzymatic reaction to resemble a continuous plug-flow bioreactor configuration. From a bioreaction engineering aspect, an ideal plug flow reactor has the same performance equation as a batch reactor. This concept was utilized in the standardized semi-dynamic protocol by transferring portions of ongoing gastric-phase digesta into several batches of static intestinal phase.

Nutrient absorption by intestinal cells can be simulated by using a membrane filtration unit. Incorporation of nutrient absorption process in a dynamic model enables the continuous removal of enzymatic hydrolysis products. Continuous product removal eliminates the effect of product inhibition on intestinal digestive enzymes, particularly trypsin and chymotrypsin, which were reported to exhibit product inhibition [62–65]. Based on the *in-vivo* data of blood nutrient concentration, the nutrient absorption process in a dynamic *in-vitro* model can be designed using engineering analysis of mass transfer [66–68].

Several authors have modeled the intestinal digestion process [69–72]. Pompa et al. [73] employed reaction engineering approach by considering intestines as sequences of continuous stirred tank reactor coupled with membrane filtration unit. There are three type of intestinal movement, namely peristaltic, segmentation, and pendular movement. The significance of each intestinal motility and villi structure on the nutrient flow and mass transfer has been studied in details by several authors [70,74,75].

Recent advances in clinical technologies, such as such as Positron Emission Tomography (PET) or MRI scan have enabled the measurement of the several engineering parameter in an *in-vivo* condition. Imaging technologies of gastric and duodenum were reviewed by Schulze [76] MRI scan provides real-time high-resolution imaging of gastrointestinal anatomy. This technique allows the quantification of parameter such as gastric juice diffusion to the meal [77]. Moroever, it allows the validation of computer simulation of gastric flow and mixing [78], and study of the encapsulated particle behavior [79]. PET employed tracers which could be used to investigate the molecular functions. Glucose absorption could be investigated using absorbable tracers (fluorodeoxyglucose) [80], while fluid distribution in the gastrointestinal tract could be analyzed using non-absorbable tracer (Deoxyfluoropolyethyleneglycol) [81].

## 3. Application of *in-vitro* digestion model in fish

### 3.1. Current *in-vitro* digestion studies on fish

The applications of *in-vitro* digestibility assay for fish were first reviewed by Moyano et al. [20] in 2015. Since then, less than 20 new studies have been published (Table 2). Most studies have primarily focused on rainbow trout (*Onchorychuss mykiss*), about 30% of the total studies using *in-vitro* digestion for fish), followed by several other species such as bream (*Sparus aurata*), tuna, tilapia, Atlantic salmon, and carp (Table 3). Trout is indeed one of the most economically important fish, second only to carp [82]. Other high-economic species are tilapia and catfish species. However, a significantly less amount of study has been done on other economically important species such as carp and tilapia. One reason for the high *in-vitro* digestibility study on rainbow trout compared to carp and tilapia is that rainbow trout, is a carnivorous fish. Carnivorous fish require food with higher protein content compared to herbivorous fish. Because protein is the most expensive and difficult ingredient to be replaced, research on developing fish meal replacement, using the *in-vitro* method, primarily targeted for carnivorous fish. Several other fish species have also been studied based on their local economic importance [7,16,83–85].

**Table 2.** Recent *In-vitro* digestibility assay performed on fish

pH	E:S ratio (U/mg protein)	T°C	Time (min)	Ingredient	Measured parameter	Species	Note	Ref
2 & 8	-	25	60 & 60	24 protein ingredients	DH	*Onchorychus mykiss, cobia, tilapia*	Two stage pH stat. Compare caged and farm tilapia	[136]
2 & 8	12.5 & 16.7	37	60 & 180	Fish and soybean meal	Peptide fraction, FAA Residual protein	Pacific bluefin tuna	Two stage digestion, with commercial pepsin and fish crude intestinal extract	[146]
2 & 8	12.5 & 16.7	37	60 & 180	Fish and poultry meal	Peptide fraction, FAA Residual protein	Pacific bluefin tuna	Two stage digestion, with commercial pepsin and fish crude intestinal extract	[147]
8		37	60	Soybean meal on different composition with fish meal	DH, pHdrop, zymography, APD	*Mystus nemurus*	One stage intestinal digestion with 4 enzyme system: fish crude extract and 3 mammalian enzymes mixture	[84]
3 & 8	24.125 & 2.875	37	15 & 45	13 protein ingredients	DH FAA Zymography	*Lutjanus guttatus*	One and Two stage pH stat. Comparison between two fish developmental stage	[166]
3.5 & 8	6.25	37	15 & 45	Soybean concentrate on 7 different level	DH, Nutrient retention (N, P), Invivo	*Totaba magnodali*		[83]
8	nd	25	60 min	*Nannochloropsis granulata*	DH	*Oncorhynchus mykiss* *Litopenaeus vannamei*	Evaluate different treatment by supercritical CO_2_ extraction	[167]
3.5–6.5 & 6.5–8.5	125–500 & 50–200	nd.	240–480 & 480–640	Comerical feed	FAA Reducing sugar	*Sparus aurata*	Two stage membrane reactor, with fish crude extract	[23]
3.5 & nd.	nd.	37	15	11 protein ingredients	DH, FAA APD	*Centropomus undecimalis*	Two stage pH stat	[87]
8	-	25	60	10 protein ingredients	DH, invivo	*Acipenser baeri*	One step intestine stage,	[168]
3.4&7.8	256 & 1460	28	90 acid	Poultry by product on several different grade	Pepsin assay FAA APD	*Lates calcarifer*	Two stage membrane reactor, with commercial pepsin and fish crude intestinal extract	[169]
9	2.5	25	90	4 microalgae	SDS page, FAA	*Senegalese sole*	One stage intestinal digestion	[170]
2 & 7	nd	37	120 & 120	Three commercial diet	In vivo assessment Residual protein Minerals (P, Fe, Zn)	*P.mesopotamicus*	Two step digestion with final product dialysis separation. Added bile extract	[16]
3.5 & 8	-	-	15&45	16 protein ingredients	DH, FAA	*Amphilophus trimaculatus*	Intestinal extract	[90]
2.1 & 8	-	-	60 &240	Two mixed diet	Minerals	*Onchorychus mykiss*	Intestinal extract Compared with and without inorganic supplementation	[171]
nd	13.02 &11.27	37	nd	Soybean β-conglycinin	SDS page, FAA, qPCR,	*Ctenopharyngodon idella*	Stomach and pyloric extract	[143]
8	nd.	37	10	Fish, soybean, and squid meal	pH drop	*Mystus nemurus*	One stage intestinal digestion with 4 enzyme system: fish crude extract and 3 mammalian enzymes mixture	[85]
The notation ‘&’ signifies pH, ES ratio, and duration of gastric and intestinal phase. Data without notation ‘&’ indicate only single intestinal phase. nd. = not determined FAA for free amino acids; DH for pH-stat degree of hydrolysis; APD for *in-vivo* based apparent protein digestibility.

**Table 3.** *In-vitro* digestibility assay on different species of fish. Coded references are listed in Supplementary references

Species	Reference	Count
Trout	5 6 9 16 21 22 23 24 30 32 33 38 40 41 47 55	16
Larvae	8 11 12 14 28	5
Bream	26 31 34 36 48	5
*Salmo salar*	10 19 25 20 39	5
Tuna	7 35 42 43	4
Tilapia	3 18 37 41	4
Carp	1 2 32 56	4
Catfish	44 57	2
Flatfish	4 13	2
Other cichilid	54	1
Pacu	53	1
Barramundi	51	1
Siberian Sturgeon	50	1
Snook	49	1
Totoaba	46	1
Snapper	45	1
Cobia	41	1
Meagre	37	1
European bass	37	1
Cod	27	1

Several protein ingredients have also been evaluated using an *in-vitro* fish digestion model. Fish meal stands at the top ingredient as it is the current protein source for fish feed to be used as the control despite several studies have used casein as the standard (Table 4.). It is sure enough that soybean is the second protein ingredient studied most by *in-vitro* digestion, given its global recognition as high protein feed material. By using an *in-vitro* digestion model, several studies have evaluated the effect of inhibitors and phytic acid on the digestibility of soybean to be utilized as a fish meal substitute [86–88]. Substitution of fish meal with soybean formulated as a fish feed had been studied on several different levels ranging from none to total replacement [83,84].

**Table 4.** *In-vitro* digestibility assay on different protein ingredient. Coded references are listed in Supplementary references

Feed	Reference	Count
Fish meal	2 5 6 9 10 11 13 16 18 19 20 23 25 27 28 30 33 34 35 36 38 41 42 43 45 50 54 57	28
Soybean meal	2 5 9 13 16 18 19 27 28 32 34 36 38 41 42 44 45 46 49 50 54 56 57	23
Mixed diet	7 11 12 14 15 17 20 26 29 31 37 39 40 48 53 55	16
Casein	2 9 11 13 16 24 28 34 36 45 54	11
Poultry (byproduct) meal	9 21 27 41 43 45 49 50 51 54	10
Corn gluten	5 9 13 27 34 41 45 49 50 54	10
Wheat gluten	9 16 27 28 41 45 49 50 54	9
Meat meal	16 18 19 34 45 50 54	7
Squid meal	11 13 28 45 54 57	6
Rapeseed meal	27 41 45 49 50	5
Other seeds*	2 18 19 27 41	5
High-starch meal*	5 9 18 41 54	5
Hydrolyzed feather meal	9 27 41 50 54	5
Blood meal	34 41 50 54	4
Krill meal	13 27 28 45	4
Algae	1 47 52	3
Single-cell protein	1 22 50	3
Shrimp meal	18 27 54	3
Peameal	13 27 32	3
Zooplankton	8 28	2
Haemogoblin	45 54	2
Dried whey	11 49	2
Mussel and crab meal	13 27	2
Lupin meal	27 34	2

The use of the *in-vitro* digestibility method to evaluate the novel ingredient for fish meal replacement is limited; only five studies had evaluated algae and other single-cell proteins (Table 4). To the authors’ knowledge, no study has been conducted using a fish *in-vitro* digestion model to evaluate the protein digestibility of an insect meal. Given the wide range of species for insects, algae, bacteria, yeast, or edible filamentous fungi, together with the combination of different cultivation techniques, and the ingredients post-processing, the *in-vitro* digestibility method offers peculiar advantages in screening numerous variables before a lengthy *in-vivo* experiment is conducted.

Only two studies have assessed the *in-vitro* digestibility of lipids [89,90] and carbohydrates [91] of fish feed. Protein indeed is the main cost of a fish feed ingredient. However, lipid requirement is crucial in fish survival, particularly phospholipid during the larval stage [92,93]. The small size of fish larvae is a particular challenge for *in-vivo* assessment of nutrient digestibility; thus, *in-vitro* approaches offer a prospective alternative to develop fish larvae feed.

### 3.2. Fish *In-vitro* Gastrointestinal Model

The fish gastrointestinal tract is commonly partitioned and termed as the headgut, foregut, midgut, and hindgut. The headgut includes the mouth and pharynx. The foregut includes the esophagus and stomach; midgut and hindgut refer to the small and large intestine compared to humans. Between the stomach and midgut, there is an anatomical feature called pyloric ceca, where the pancreatic enzymes are secreted.

The main difference in the *in-vitro* digestion study on fish and humans is the source of enzymes. Since there are no commercial fish digestive enzymes, it is required to extract the digestive enzymes from the fish itself. Several studies compared the crude extract of fish digestive enzymes with mammalian enzymes, showing proteases from fish crude extract have higher degree of hydrolysis compared to mammalian enzyme [20,94,95]. However, some studies showed contradictory results [84,85,96]. There are several reasons for the inconsistency as pointed out by Moyano et al. [20], including substrates affinity, enzyme kinetics, optimum temperature and sensitivity to inhibitors. Mammalian digestive enzymes are more susceptible to product inhibition compared to fish [97]. It has been regarded by several authors that performing an *in-vitro* digestibility assay species specific digestive enzyme extracts allows a better representative with in-vivo condition [20,97,98].

In the total of 57 studies on *in-vitro* fish digestion, about 80% of the studies of fish were done in a static *in-vitro* model, and the rest were done in a membrane bioreactor model with continuous nutrient absorption. Half of the total studies employed a pH-stat method to control the digestion pH and measured the degree of hydrolysis of the protein ingredient. Based on the species and developmental stage of the fish being studied, the *in-vitro* gastrointestinal model could be a one-stage or two-stage digestion process. Some herbivorous fish are stomachless, thus skipping the acid digestion phase in the *in-vitro* model is omitted. Carnivorous fishes possess a functional stomach. However, a third of the study on carnivorous fish have skipped the gastric phase in the static digestion model [19] despite its several significant roles, such as increasing protein and mineral solubility [56,67,78], pre-digesting the proteins, and deactivating the antinutrient factor [79]. Understanding the targeted fish species gastrointestinal physiology is critical before conducting an *in-vitro* digestion study. Fish larvae have yet to develop a functional stomach; thus, it is best to simulate the intestinal digestion process in fish larvae in a one-step digestion model using the enzymes extracted from the whole larvae.

Numerous studies used a membrane reactor to simulate the nutrient absorption process by the fish intestines [91,99–101]. The membrane reactor was based on the initial design of Savoie and Gauthier [102]. This membrane bioreactor has two chambers, an inner reaction chamber, and an outer chamber, separated by a dialysis membrane with a molecular weight cutoff of 1000–3500 Da [20,102].

Currently, the concept of gradual secretion of gastric juice and gastric emptying is absent in the study of *in-vitro* digestibility for fish. The dynamic models developed for humans have potential to be adapted for fish, except the model that explicitly featured the human anatomical feature, such as the J-shaped stomach in DIVHS, GDS, and ESIN. A dynamic model such as DIDGI, TIM-1, SimuGIT, and membrane bioreactor model by [102,103] can be utilized to simulate the dynamic process of gastric digestion, including mixing, gradual gastric juice secretion, and gastric emptying. The INFOGEST semi-dynamic *in-vitro* digestion model has a high potential not only as a standardized method for humans but also for fish due to its simplicity and replicability using standard laboratory equipment. An engineering approach has benefits in tackling the issue of adapting the humans model on fish as it can objectively quantify the required change of the *in-vitro* model developed for human application based on any fish species *in-vivo* condition.

Obtaining the *in-vivo* relevant data such as chyme viscosity and particle size distribution for human is costly and challenging [50]. Therefore, study on humans relies on the usage of a pig as the model. However, acquiring *in-vivo* relevant data in fish is more accessible compared to mammals and humans. Sacrificial fish is cheaper for fish compared to mammals. In fact, several studies have used the radiography technique for the *in-vivo* study on gastric emptying of fish [104–106].

Numerous studies have highlighted the importance of gut microbiota on fish health as an immunostimulant. Prebiotics, mainly as the non-digestible polysaccharide, directly affect the fish gut microbiota [107–110]. Current microbiota studies on fish rely on the genomic and metagenomic analysis of the fecal sample; however, this method is costly, both from financial and temporal standpoints [111]. Application of an *in-vitro* model, such as SHIME and SIMGI has high potential to investigate the effect of fish feed on the gut microbiota.

### 3.3. Challenges in fish *in-vitro* digestion study

Conducting an *in-vitro* digestibility of a fish feed means mimicking the digestion processes along the gastrointestinal tract: enzymatic digestion in the fish stomach and intestine; in a laboratory. First, the required enzymes are extracted from fish digestive tissues (stomach and *pyloric ceca*). The fish feed, and the enzymes are then added to a gastrointestinal model. The amount the enzymes added should simulate the *in-vivo* condition in the fish; hence, it is crucial to have the information on the enzymatic activity of the enzymes successfully extracted. The digested sample is then measured using several analytical techniques. For the validation of the *in-vitro* gastrointestinal model, the result is compared with the *in-vivo* study. Each step of the process possesses a unique challenge that the current *in-vitro* digestion model is facing.

#### 3.3.1. Sources of Enzymes

One of the challenges in conducting an *in-vitro* digestion study for fish is sourcing the digestive enzymes. Fish secretes the same enzymes as in mammals or humans, including gastric pepsin, pancreatic trypsin, chymotrypsin, amylase, and lipase. However, there are two problems. First, fish species have different catalytic properties compared to the mammalian analog. For example, turnover number (k_cat_) and Michealis constant (K_M_) of trypsin from rainbow trout are 1.7–3.28 s^−1^ and 54–77 µM, compared to 0.7–1.63 s^−1^ and 342–455 µM on bovine trypsin at a temperature ranging from 10–20°C [112].

Second, the amount of digestive enzymes secreted by fish is highly dependent on the environmental and nutritional factors, as recently reviewed by Lallès [113], causing high variation between different studies. Water acidification (from pH 8 to 7.5) and rearing temperature (18°C to 2°C) could decrease the intestinal protease activity by 36% and 56%, respectively [113]. Water salinity and oxygen level could also significantly affect the alkaline protease activity. The effect of fish rearing nutrition (protein and lipid source, carbohydrate, lipid source) on protease activity has been reported [113].

Nearly all of the *in-vitro* digestion studies on fish obtained the digestive enzyme from an in-house reared fish under controlled conditions. Fish was reared by feeding with a commercial diet until the age of interest to be modeled. Fish is then starved before being sacrificed. The stomach, *pyloric ceca*, and intestines were then dissected and extracted for the digestive enzymes. This method ensuring a lower variability in the culture condition on affecting the amount of digestive enzyme secreted, however, it takes a relatively same lengthy process to do an *in-vivo* study.

#### 3.3.2. Enzyme Extraction

Fish digestive enzymes could be extracted by homogenizing the digestive tissue in a liquid media, followed by centrifugation to remove the tissue debris. The gastric and intestinal protease extraction method from fish digestive tissues was recently reviewed and studied [114,115]. It is recommended to use distilled water acidified to pH 2 to extract pepsin from the fish stomach, yielding three times the enzyme activity compared to other media, such as distilled water, buffer solution, and 25 mM NaCl [115]. Whereas for intestinal protease from the *pyloric ceca* or intestinal enzymes, Tris buffer (pH 8) is recommended [114]. The volume of liquid media to tissue ratio ranges from 1:1 to 1:10 in a single-step extraction or multiple-step extractions of 1:3 w/v [98,115,116]. The centrifugation process is carried out to remove the insoluble solid debris. The supernatant, denoted as the crude extract, is then used as the digestive enzymes added into the gastrointestinal model for the *in-vitro* digestibility assay.

The amount of crude extract added to the digestion process depends on the crude extract enzymatic activity, keeping the same enzymatic activity throughout the study. Since the crude extract is highly diluted, the difference in the volume of crude extract added to maintain constant enzymatic activity causes significant dilution in the digestion system, resulting in a slower digestion rate as enzymatic catalysis is highly dependent on the substrate concentration [99]. Only a few studies processed further the crude extract. One author had concentrated the enzymes by freeze-drying without any purification [117,118]. Three studies purified the crude extract using Brij 35 extraction, ammonium sulfate precipitation, and chromatography separation (gel and affinity chromatography) [98,116,119]. However, it must be noted that if the crude extract needs to be further purified, distilled water should be used as extraction media instead of HCl or buffer as the concentration will increase the acid and buffer concentration which could have an adverse effect on the protease.

Storage of the extracted enzymes is one of the critical factors that has been neglected. Protease (pepsin, trypsin, and chymotrypsin) activities in a crude extract decreased over time even stored at −20°C had shown to affect the enzyme activity over time [120,121]. Freezing of enzymes could cause the reduction of pancreatic enzyme activity by half in one thawing cycle [121]. Only one study reported the stability of enzymatic activity throughout the storage period [116].

Several improvements can be made on sourcing and extracting the fish digestive enzymes. The extracted enzymes can be either concentrated or purified further, limiting dilution when adjusting the required enzymatic activity. In this way, the enzyme secretion variability due to the rearing condition can be resolved. Fish gutting waste from industry or local market can be used instead of in-house fish rearing, following the commercial production of porcine/bovine pepsin, trypsin, or pancreatin from slaughterhouse by-product. Several studies have elaborated the downstream processing for the fish gutting waste enzymes recovery with commercial purpose instead of utilization for *in-vitro* digestion study [122,123].

#### 3.3.3. Determination of Enzymatic Activity

The amount of crude extract added to the *in-vitro* gastrointestinal model need to represent the *in-vivo* condition. Therefore, it is necessary to measure the enzymatic activity of the crude extract. Several methods to determine the fish pepsin activity were reviewed in detail by Nolasco‐Soria et al. [115]. The method to measure protease activity was proposed by Anson in 1938 [124], using hemoglobin as the protein substrate and folin-ciocalteu reagent for the spectrophotometric assay of hydrolyzed products. Since then, several modifications have been made to improve the original method. However, it is still the most-cited method for quantification of acid pepsin in fish [115]. Moreover, a recent standardized protocol on *in-vitro* digestion for humans quantifies the pepsin activity based on Anson’s hemoglobin substrate method with modification of using UV-spectrophotometric measurement at λ = 280 nm instead of using a folin reagent [24].

For the quantification of intestinal protease in fish, the most cited method is developed by Walter [125], using casein as the substrate and measurement using UV-spectrophotometer at λ = 280 nm [114]. A recent review and investigation by Nolasco‐Soria [114] suggested using casein as substrate, followed by *o*-phenylenediamine spectrophotometric assay. Intestinal protease consists of trypsin, chymotrypsin, elastase, and carboxypeptidase. Instead of quantifying all intestinal protease activity, individual intestinal enzyme activity present in alkaline proteases, such as trypsin or chymotrypsin, could be determined using synthetic chromogenic substrates. Several substrates such as BAPNA (benzoyl dl-arginine p-nitroanilide hydrochloride) and TAME (p-toluene-sulfonyl-L-arginine methyl ester) could be used for determining trypsin activity. BAEE (N-benzoyl-L-tyrosine ethyl ester) has been utilized to quantify chymotrypsin activity [126]. In fact, TAME was used in the standardized *in-vitro* method on humans to quantify pancreatin enzymatic activity [24].

#### 3.3.4. Operating Condition

The protein digestibility of an ingredient is affected by two factors: intrinsic and external factors. An *in-vitro* method is developed as a cheaper and faster alternative in assessing those factors to the *in-vivo* method. However, in the context of *in-vitro* digestibility, another factor that affects protein digestibility is the method itself, either the digestion model or the operating parameter. Several operating parameters affecting the *in-vitro* digestibility include digestion temperature, gastric and intestinal phase pH, gastric and intestinal phase duration, the enzyme-to-substrate ratio (ES ratio) [127].

Ideally, those factors follow the *in-vivo* condition present in the fish digestive system. Gastric and intestinal pH can be measured by inserting a pH probe into freshly dissected fish digestive tissues. Temperature is determined based on the rearing temperature of the fish. Digestion duration is reflected by the duration of food in the gastrointestinal tract, termed as ‘gut transit time,’ which could be calculated based on the fecal analysis [23,128] or by direct measurement of the gut content of the fish fed by dyed marker [117]. The ES ratio is calculated based on the measured total enzymatic activity in fish digestive tissue, and the fish feed ingested [99].

**Figure 3.**
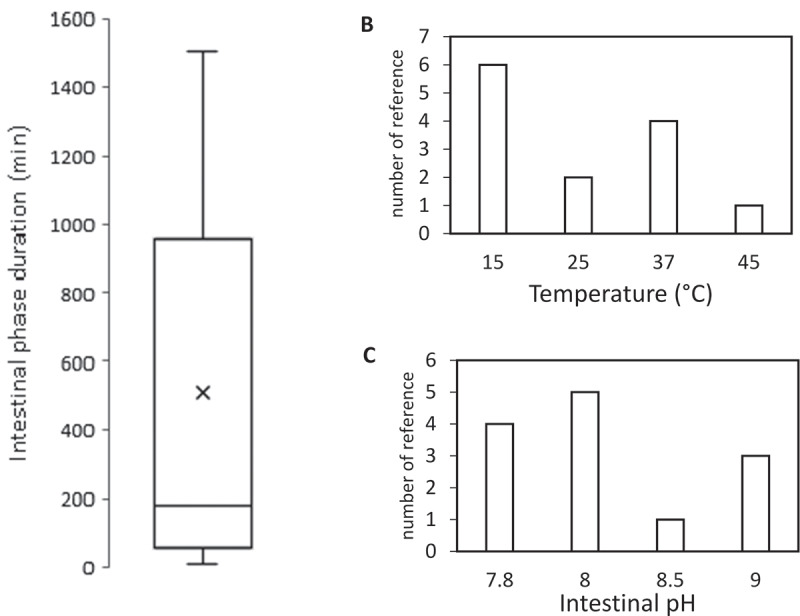
Employed operating condition throughout 16 reviewed studies of *in-vitro* digestion for *Onchorychus mykiss.*

However, those variables vary for different fish species, growing stages, etc. The stomach and intestinal pH were shown to fluctuate along with the duration of the day [129]. Rearing temperature is highly affected by the season and geography. In addition, the number of secreted enzymes varies highly by numerous factors, as pointed out in the previous section. Digestion duration is affected by the feeding frequency [128], rearing temperature [105,130], and the structure and particle size of the feed [131,132]. For those reasons, the selection of operating conditions for *in-vitro* study based on the *in-vivo* condition is highly variable between studies, even for single species, such as Rainbow trout, as shown in Figure 3.

A good *in-vitro* method should be robust and provide a consistent conclusion when replicated by other laboratories with minor variation. Therefore, it is important to identify the possible variations of the method, which could significantly affect the result [133]. Factorial design experiments could be employed to assess this issue. Factorial design was conducted to study the interaction between the aforementioned operating variables showed that, there are interactions between ES ratio with pH and digestion duration [23,127]. Tibbetts et al. [116] reported an optimal ES ratio between 7 and 30 BAPNA units per milligram substrate protein on the *in-vitro* digestion study for Atlantic cod. However, a similar study has not been performed for other fish species.

Since fish encompasses a vast number of species, it is extremely difficult to follow the human approach to standardize the operating condition by an international consensus. An alternative approach is proposed by selecting operating conditions, such as optimum pH and ES ratio, based on their optimum value. However, the optimum temperature of fish (rainbow trout) trypsin could reach up to 60°C [112] and does not represent the *in-vivo* condition. Therefore, the selection of operating temperature could be based on the fish rearing temperature. The protein digestibility is preferably reported in a time profile of the digestion process until reaching the steady-state; however, this imposes an extra experimental cost in the number of analyzed samples.

There are still several factors that have not been included in the study of *in-vitro* digestion of fish, such as the effect of bile, electrolytes, and particle size. Bile affects lipid digestion, and it is known that lipid could interact with protein hydrophobic molecules or as a lipoprotein. Only one study incorporated the bile salt in the study [91], showing a two-fold increase of protein digestibility with it. Electrolytes are known to affect protein solubility but have not been systematically studied for *in-vitro* digestion on fish. The electrolyte concentration in the fish gastrointestinal tract could be measured as the procedure elaborated in Becker et al. [134] and recreated using inorganic salts.

#### 3.3.5. Analytical Method

**Figure 4.**
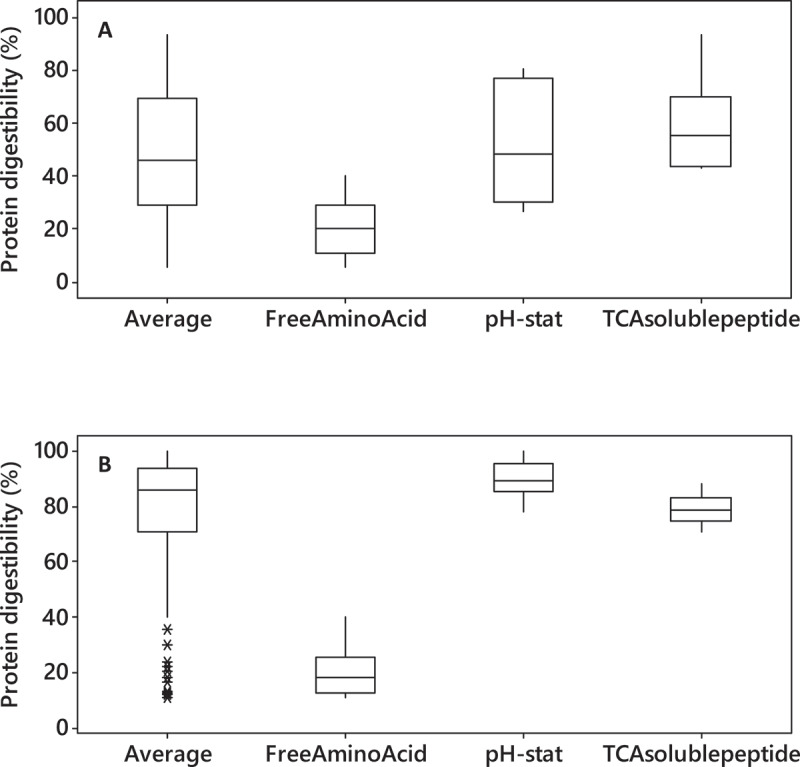
(a) Soybean (b) fish meal *in-vitro* protein digestibility using different analytical method

The *in-vitro* protein digestibility of soybean and fish meal are averaged at 50% and 85%, respectively, from the 57 reviewed articles, but they are highly dependent on the measurement method (Figure 4). The selection of the analytical method is crucial to obtain a reliable conclusion on protein digestibility. Several analytical methods widely used to assess protein digestibility include the pH-stat method, free amino group measurement, TCA-soluble protein, and peptide distribution by either SDS-PAGE or size-exclusion chromatography.

##### 3.2.5.1 pH-stat Method

The pH-stat measurement is a classical approach to monitor the enzymatic digestion process by titrating acid/base to maintain constant pH. The degree of hydrolysis (DH) can be calculated based on the volume of acid/base added by:

**Table:** 

Acid (pH ≤ 5):	$DH=100x\frac{V\times N}{m\times h_{tot}}\times \frac{1}{1-\alpha_{COOH}}$	$\alpha_{COOH}=\frac{10^{pH-pK_{a}^{COOH}}}{1+10^{pH-pK_{a}^{COOH}}}$	(1)
Neutral/Basic (pH ≥ 7) :	$DH=100x\frac{V\times N}{m\times h_{tot}}\times \frac{1}{\alpha_{NH_{2}}}$	$\alpha_{NH_{2}}=\frac{10^{pH-pK_{a}^{NH_{2}}}}{1+10^{pH-pK_{a}^{NH_{2}}}}$	(2)

V is the volume of titrant (mL); N is the normality of titrant; m is the protein mass (g); h_tot is_ the number of peptide bonds per gram of proteins. α_COOH_ and α_NH2_ are the degree of dissociation of carboxylic and amino groups produced, estimated from the pKa of the carboxylic and amino groups [135].

Several studies tried to correlate the DH value with several other measurements, such as *in-vivo* protein digestibility as summarized in Table 5. It has been regarded as a valuable approach for conducting an *in-vitro* digestion experiment, providing several undeniable advantages in real-time monitoring, easy to set up, and a nondestructive way to monitor and evaluate the kinetics of enzymatic hydrolysis of proteins ingredients. However, the pH-stat method seemed to be highly inconsistent. Moreover, it was reported that the DH value of conducting *in-vitro* digestion using a single gastric phase could be two times higher than the combined gastric and intestinal phase for a particular protein ingredient [136], but the reason was mainly not explained. A possible reason for the variation between studies is the assumption of α value, which is dependent on temperature and the protein ingredient (peptide chain length and terminal amino acid) [137]. Another reason is the buffering capacity of the feed ingredient, which has been reported to severely affect the pH-stat method [101]. Moreover, titration with NaOH during the intestinal phase also measures the lipid hydrolysis of the ingredient.

**Table 5.** Correlation of pH-stat method with *in-vivo* protein digestibility

Species	Correlation Equation	R^2^	Notes	Reference
Rainbow trout	y = 1.98 x + 39.4	0.64	Mammalian enzymes	[98]
	y = 1.67 x + 41.2	0.82	Trout enzymes	
Gadoids	y = 1.30x + 70.57	0.34	Recalculated from the provided data	[119]
Catfish	y = 1.33x + 29.28	0.930	Fish enzymes	[84]
	y = 1.05x + 56.21	0.853	Single enzyme	
	y = 1.07x + 33.56	0.895	Three enzyme mixture	
	y = 1.54x + −17.66	0.924	Four 4 enzyme mixture	
*Totaba magnodali*	y = 12.59x – 1.8018	0.792	Recalculated from the data	[83]
Siberan Sturgeon	y = 10.62x + 32.08	0.879	Animal based meal	[168]
	y = 6.627x + 43.531	0.967	Plant based meal	
	y = 1.30x + 70.57	0.34	Overall	
Snook, *Centropomus undecimalis*	Y = 1.8968 x + 88.52	0.73	Fish digestive enzyme for 11 ingredient	[87]

##### 3.2.5.2 TCA-soluble peptide

The measurement of digested protein is based on the assumption that undigested proteins with high molecular mass are precipitated either by Trichloroacetic acid (TCA) or perchloric acid. Precipitated proteins are then removed by centrifugation, leaving only the digested peptides in the supernatant measured for the protein content. Measurement of protein content was reviewed in detail by Hayes [138]. The Kjeldahl method is considered the global standard method for protein measurement of food products, particularly for a solid sample. The spectrophotometric method includes biuret methods (Lowry and BCA), Bradford assay, and UV spectroscopy are fast, easy, cheap, and able to measure small amounts of protein. However, it is only suitable for liquid samples [138]. The known golden standard (Recommended by FAO) of protein measurement is the direct amino acid measurement [138]. It is not only accurate but also provides information on the essential amino acids. The drawback of this method is that it is time-consuming and requires the installation of an expensive instrument such as HPLC, UPLC, or LC-MS.

##### 3.2.5.3 Primary Amino Group

Protein is hydrolyzed into peptides and amino acids, which possess a primary amino group at the end of a peptide. Several reagents could react specifically with only primary amino groups, including *o*-phenylenediamine (OPA), ninhydrin, Trinitrobenzenesulfonic acid (TNBS), and fluorescamine. OPA method is the most widely used in the study of *in-vitro* digestion compared to others. The OPA method was first introduced by Church et al. [139] to measure the degree of hydrolysis of milk proteins [140].

The measurement of primary amino groups could be either termed as both free amino acid measurement or degree of hydrolysis. The former is commonly termed in the study of *in-vitro* digestion on fish, despite a single molecule of primary amine present in dipeptide, oligopeptide, or polypeptide. The term degree of hydrolysis is more appropriate as mentioned in the original method [137,139,141] and most of the *in-vitro* digestion study for humans. The value of the degree of hydrolysis by this method is used to calculate the pH-stat method α value [137].

##### 3.2.5.4 Protein and Peptide Fractions

A protein ingredient commonly includes several different proteins. SDS-PAGE analysis provides valuable qualitative information on which proteins are digestible or resistant to enzymatic hydrolysis at each digestion phase. Sousa et al. [17] studied the *in-vitro* digestion of different plant protein sources, identifying several proteins resistant to either the gastric or intestinal digestion phase. Manditsera et al. [142] evaluated the effect of several cooking methods on the protein digestibility of insects. They found that cooking by boiling increases the digestibility of high molecular weight protein by denaturation. Despite its widespread usage for mammals, only four studies have reported SDS-PAGE usage to visualize the digested protein molecular weight distribution in studying *in-vitro* digestion for fish [143–145]. The study by Duan et al. [143] on β-Conglycinin, a storage protein found in soybean, found that the alpha subunit is highly digestible while the beta subunit is resistant in the *in-vitro* digestion process of fish.

The SDS-PAGE result can be analyzed qualitatively via visualization of the protein bands or quantitatively by scanning the gel in a densitometer. The image is then analyzed by an image processing software provided by the densitometer, or open-source software, such as ImageJ. Based on the optical density, the Coefficient of protein degradation could be calculated as described by Alarcon et al. [145].

Another method to measure protein fraction quantitatively is by size-exclusion chromatography. This method employs HPLC columns that separate molecules based on the molecular weight. Only four studies had employed this analytical technique [146,147]. Using this technique, the author showed that *in-vitro* digestion of soybean minimally increases the low-molecular-weight peptides (<6.5 kDa) compared to fish meal, while similar molecular weight distribution between hydrolyzates of fish meal and poultry meal was observed [117,118,146,147] . *In-vitro* digestion of single-cell protein using rainbow trout digestive enzymes yielded two times higher of products with molecular weight between 250 and 4000 Da compared to products with molecular weight less than 250 Da.

##### 3.2.5.5 Microscopy

Microscopy technique is known to visualize the structure of the solids particle, protein aggregates, or lipid emulsion for *in-vitro* digestion study. This technique provides qualitative information on the effect of the microstructure of the food on enzymatic hydrolysis. For novel feed ingredients with recalcitrant cell-wall structure, the technique provides an analysis of cell wall integrity and the release of cellular components during the digestion process [148]. There are several different types of microscopy techniques to visualize food structure, including optical, fluorescence, and confocal microscopy. To observe the molecules of interest, several stains could be employed, for instance, Fluorescein isothiocyanate [148], Commasie, Fast green [148], Evans’s blue for staining proteins [148], Sudan black for staining lipids, Lugol for carbohydrate, Toluidine blue for nucleic acids, Calcofluor white or cotton blue for cellulose or chitin present in the plant cell wall.

The microscopy techniques are widely used in *in-vitro* digestion for humans. Colosimo et al. [148] showed that the mycoprotein treated by ultrasonication increased the protein digestibility by increasing the porosity of the cell wall, enabling the digestion inside the cell despite the intact cell wall. Using confocal microscopy, the study of soybean milk digestion showed that as the protein aggregates are partially hydrolyzed during the gastric phase, the peptides on the protein surface exhibit surface-active agent properties, attracting the lipids and covering the protein aggregate [149]. The finding proves the crucial role of lipase in the *in-vitro* digestion protocol. Despite its common application for the study of *in-vitro* digestion for humans, the procedure is never used to study *in-vitro* digestion for fish. The knowledge on the microstructure could be beneficial for the research on the improvement of novel protein ingredients for fish meal replacement.

#### 3.3.6. Comparison of *in-vitro* and *in-vivo* methods

The result of an *in-vitro* model must be validated with an *in-vivo* experiment. There are several methods to gather the *in-vivo* data of protein digestibility in fish. Apparent protein digestibility (APD) is the most measured variable by collecting the fish fecal sample. The term ‘apparent’ denotes the potential of both overestimation and underestimation from ‘true’ protein digestibility [150]. This method is the most common to validate the *in-vitro* experiment on fish; however, a systematic discrepancy will be observed as the current *in-vitro* method excludes the fermentation by microbial community in the distal intestine.

Another more detailed method to evaluate the *in-vivo* protein digestion collects the samples inside the fish gastrointestinal tract after a certain feeding period, as suggested by previous review [20]. The collected samples could be analyzed for their protein content, peptide molecular-weight distribution, or other nutrient digestibility. This protocol was commonly used to validate the *in-vitro* digestion in humans using pigs as the model. However, this method requires some sacrificial fishes. Alternatively, a direct blood measurement was utilized to measure the nutrient bioavailability in the fish [151,152].

## 4. Suggested Protocol for *in-vitro* digestion study in fish

**Figure 5.**
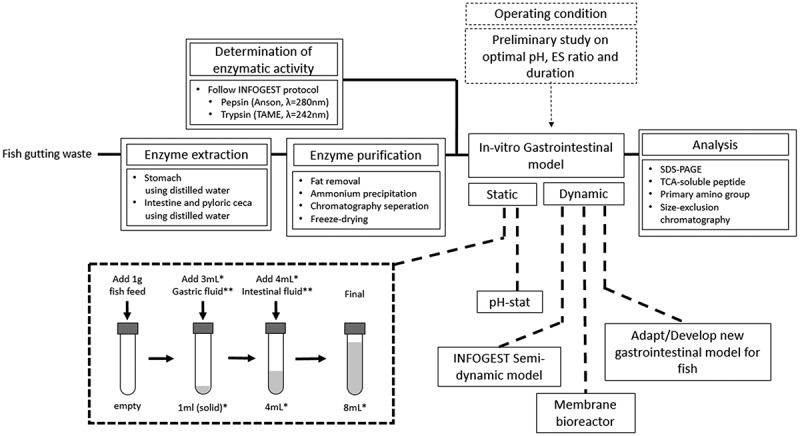
Suggested protocol for *in-vitro* digestion study for fish. *the total volume and amount of digestive fluid added could be scaled with the amount of sample. **the digestive fluid contains the amount of enzymes determined during the preliminary study

As both humans and fish share a rather similar gastrointestinal tract, several developed protocols and gastrointestinal models applied for humans could be adapted for fish. Therefore, *in-vitro* digestion protocols for fish are suggested. Based on the research aims – screening, in-depth analysis, or validation with an *in-vivo* experiment – the protocols are differentiated as static and dynamic protocols. The protocol is summarized in Figure 5.

First, the fish digestive enzymes are extracted from either in-house reared fish or fish gutting waste. Distilled water is used as the extraction media is employed to extract both the pepsin and intestinal protease, with the tissue-to-media ratio is 1:10 w/v. Then, emulsified lipid in the crude extract is removed by chloroform extraction, followed by enzyme concentration by freeze-drying, yielding the enzyme concentrate. The pepsin and trypsin activities of the enzyme concentrate are measured as in the INFOGEST protocol using hemoglobin and TAME as the substrate, respectively.

The static digestion protocol is adapted from the INFOGEST protocol, with the exclusion of the oral phase. For the fish species with a functional stomach, gastric digestion is conducted by adding 1 gram of the sample into a test tube and mixed with acidified water, acidified to pH ranging from 1 to 3 using HCl, and stomach enzyme concentrate for the duration of up to 6 hours. The amount of water and gastric enzyme concentrate required is based on the measured enzymatic activity, ranging from 1 to 200 U/mg protein, to reach the gastric-phase volume of 4 mL. After the gastric phase, the pH is adjusted to 7–9 with NaOH, then the intestinal enzymes concentrate, ranging from 1 to 200 U/mg protein, and water is added to reach the final intestinal-phase volume of 8 mL. The intestinal-phase digestion duration could be up to 6 hours. The reaction vessel and the total volume of each digestion phase could be scaled with the amount of sample added depending on the enzymes and sample availability.

Prior to the primary experiment, several preliminary experiments are required to determine the exact operating condition. The gastric and intestinal phases pH are determined based on the proteases optimal pH by varying the pH of enzymatic activity measurement, using hemoglobin and TAME as substrate, respectively. The optimal amount of enzyme is decided based on the optimal ES ratio by varying the amount of enzyme concentrate added during gastric and intestinal phases, measuring the DH either by pH-stat, TCA-soluble peptides, or OPA primary amino group. The duration of each phase is chosen based on the time required to reach a steady state.

Since there is currently no dynamic *in-vitro* gastrointestinal model for fish that could simulate the dynamics of the gastric phase and continuous nutrient removal, There is a potential to develop a dynamic model specifically for fish. Otherwise, the dynamic protocol could be based on the developed gastrointestinal model for humans. One of the promising models, which could be adopted for fish, is the INFOGEST semi-dynamic model, using a pH-stat set-up and a paddle agitator for the gastric phase, while periodically transfer the gastric digesta to several batches of intestinal phase.

Several analytical methods employed in the *in-vitro* digestion study for humans can be adopted to study fish. Current SDS-PAGE usage is still minimal on fish despite the extensive usage in the study on humans. Microscopy is also an advantageous technique that can be utilized, particularly for novel protein ingredients. These analytical methods could be either employed both on the *in-vitro* study and during *in-vivo* validation. Analysis of the digesta biochemical, physical properties, and the blood plasma nutrient concentration, the mechanism of the digestion and absorption process can *in-vitro* be validated. The limitation of this protocol is only suitable to assess nutrients digestibility in the upper gastrointestinal tract (stomach and small intestines), while the assessment of the impact of the feed sample on gut microbial community is not included.

## 5. Conclusion

Digestive systems in human, animals, and fish are biological reactors and membranes to digest food and extract nutrients. This *in-vitro* approach offers a faster and cheaper way to assess nutrient digestibility. *In-vitro* digestion models for humans have been developed to a great extent, including standardization of static models and several bioreactors-based dynamic models. On the other hand, the *in-vitro* digestion model for fish is significantly less developed. Given that both human and fish shares a monogastric gastrointestinal tract, engineering analysis is one of the approach to adapt the model for human to fish. The main differences of digestion physiology between human and fish are the enzymes biocatalytic properties, causing one of the challenges in the sourcing the enzymes. Other challenges include the determination of enzymatic activity, selection of operating condition, analytical methods, and validation with in-vivo experiment. To improve the current *in-vitro* digestion protocol for fish, a protocol based on the *in-vitro* digestion study for human is suggested.

## Supplementary Material

Supplemental Material
