# Supplementary material for: In-vitro digestion models: a critical review for human and fish and a protocol for in-vitro digestion in fish
Source: Bioengineered. 2021 Jun 30;12(1):3040–64. doi: 10.1080/21655979.2021.1940769 (PMC8806420; doi:10.1080/21655979.2021.1940769)
Supplement: Supplemental Material [file KBIE_A_1940769_SM5054.zip › supplementary/Supplementary Reference File_Updated.docx]

**References for the coded number in Figure 1.**

|  | Organism | Salient Feature | Year | Reference |
| --- | --- | --- | --- | --- |
| 1 | Human | General review of available model | 2006 | [1] |
| 2 |  | General review of available model | 2012 | [2] |
| 3 |  | General review of available model | 2020 | [3] |
| 4 |  | Comparison with in-vivo | 2018 | [4] |
| 5 |  | Comparison with in-vivo | 2019 | [5] |
| 6 |  | Food allergen | 2007 | [6] |
| 7 |  | Bioactives peptide | 2020 | [7] |
| 8 |  | Intestinal absorption model | 2007 | [8] |
| 9 |  | Oral delivery of lipid based drug | 2008 | [9] |
| 10 |  | Oral delivery of lipid based drug | 2019 | [10] |
| 11 |  | Emulsion stability | 2010 | [11] |
| 12 |  | Emulsion stability | 2021 | [12] |
| 13 |  | Lipid oxidation | 2020 | [13] |
| 14 |  | Gut fermentation model for Prebiotic | 2019 | [14] |
| 15 |  | Gut fermentation model for Prebiotic | 2020 | [15] |
| 16 |  | General gut fermentation model | 2016 | [16] |
| 17 |  | Application gut model on carbohydrate | 2021 | [17] |
| 18 |  | Impact of fiber on starch digestion | 2018 | [18] |
| 19 |  | Starch digestion in general | 2018 | [19] |
| 20 |  | Computational modeling for Starch digestion | 2018 | [20] |
| 21 |  | Computational modeling in general | 2017 | [21] |
| 22 |  | Structural breakdown of starch | 2021 | [22] |
| 23 |  | Bolus formation and disintegration | 2012 | [23] |
| 24 |  | Gastric mixing | 2017 | [24] |
| 25 |  | Structural breakdown during gastric digestion | 2008 | [25] |
| 26 |  | Structural breakdown during gastric digestion | 2014 | [26] |
| 27 |  | Engineering Aspect of digestion | 2016 | [27] |
| 28 |  | Potential application of in-vitro digestion model | 2018 | [28] |
| 29 |  | Summary of operating condition | 2011 | [29] |
| 30 |  | Bioaccessibility of secondary plant metabolite | 2014 | [30] |
| 31 |  | Effect of processing on legume Bioaccessibility | 2019 | [31] |
| 32 |  | Application on dairy product | 2019 | [32] |
| 33 |  | Infants model | 2017 | [33] |
| 34 |  | Effect of food matrix on nutrient release kinetics | 2017 | [34] |
| 35 |  | Biopolymer interaction | 2021 | [35] |
| 36 | Ruminant | Gas production technique | 2010 | [36] |
| 37 |  | Semi-continuous gas production technique | 2020 | [37] |
| 38 |  | Daisy (II) Incubator | 2020 | [38] |
| 39 | Aquatic species | Application in-vitro digestion in aquaculture | 2015 | [39] |

[1] J. Y. Yoo and X. D. Chen, "GIT Physicochemical Modeling - A Critical Review," *INTERNATIONAL JOURNAL OF FOOD ENGINEERING*, vol. 2, 2006.

[2] A. Guerra, L. Etienne-Mesmin, V. Livrelli, S. Denis, S. Blanquet-Diot, and M. Alric, "Relevance and challenges in modeling human gastric and small intestinal digestion," *Trends in Biotechnology*, vol. 30, pp. 591-600, 2012.

[3] C. Li, W. W. Yu, P. Wu, and X. D. Chen, "Current in vitro digestion systems for understanding food digestion in human upper gastrointestinal tract," *TRENDS IN FOOD SCIENCE & TECHNOLOGY*, vol. 96, pp. 114-126, 2020.

[4] T. Bohn, F. Carriere, L. Day, A. Deglaire, L. Egger, D. Freitas, M. Golding, S. Le Feunteun, A. Macierzanka, O. Menard, B. Miralles, A. Moscovici, R. Portmann, I. Recio, D. Remond, V. Sante-Lhoutelier, T. J. Wooster, U. Lesmes, A. R. Mackie, and D. Dupont, "Correlation between in vitro and in vivo data on food digestion. What can we predict with static in vitro digestion models?," *Crit Rev Food Sci Nutr*, vol. 58, pp. 2239-2261, 2018.

[5] D. Dupont, M. Alric, S. Blanquet-Diot, G. Bornhorst, C. Cueva, A. Deglaire, S. Denis, M. Ferrua, R. Havenaar, J. Lelieveld, A. R. Mackie, M. Marzorati, O. Menard, M. Minekus, B. Miralles, I. Recio, and P. Van den Abbeele, "Can dynamic in vitro digestion systems mimic the physiological reality?," *CRITICAL REVIEWS IN FOOD SCIENCE AND NUTRITION*, vol. 59, pp. 1546-1562, 2019.

[6] F. J. Moreno, "Gastrointestinal digestion of food allergens: Effect on their allergenicity," *BIOMEDICINE & PHARMACOTHERAPY*, vol. 61, pp. 50-60, 2007.

[7] S. Fernandez-Tome and B. Hernandez-Ledesma, "Gastrointestinal Digestion of Food Proteins under the Effects of Released Bioactive Peptides on Digestive Health," *MOLECULAR NUTRITION & FOOD RESEARCH*, vol. 64, 2020.

[8] F. Nigsch, W. Kjaffke, and S. Miret, "In vitro models for processes involved in intestinal absorption," *EXPERT OPINION ON DRUG METABOLISM & TOXICOLOGY*, vol. 3, pp. 545-556, 2007.

[9] D. G. Fatouros and A. Mullertz, "In vitro lipid digestion models in design of drug delivery systems for enhancing oral bioavailability," *EXPERT OPINION ON DRUG METABOLISM & TOXICOLOGY*, vol. 4, pp. 65-76, 2008.

[10] R. Berthelsen, M. Klitgaard, T. Rades, and A. Mullertz, "In vitro digestion models to evaluate lipid based drug delivery systems; present status and current trends," *ADVANCED DRUG DELIVERY REVIEWS*, vol. 142, pp. 35-49, 2019.

[11] D. J. McClements and Y. Li, "Review of in vitro digestion models for rapid screening of emulsion-based systems," *FOOD & FUNCTION*, vol. 1, pp. 32-59, 2010.

[12] N. Raoufi, A. Q. Ye, and J. Z. Han, "New insights into in vivo gastroduodenal digestion of oil-in-water emulsions: gastric stability and in vitro digestion modeling," *CRITICAL REVIEWS IN FOOD SCIENCE AND NUTRITION*, 2021.

[13] B. Nieva-Echevarria, E. Goicoechea, and M. D. Guillen, "Food lipid oxidation under gastrointestinal digestion conditions: A review," *CRITICAL REVIEWS IN FOOD SCIENCE AND NUTRITION*, vol. 60, pp. 461-478, 2020.

[14] O. H. Hernandez, "In vitro Gastrointestinal Models for Prebiotic Carbohydrates: A Critical Review," *CURRENT PHARMACEUTICAL DESIGN*, vol. 25, pp. 3478-3483, 2019.

[15] L. Nissen, F. Casciano, and A. Gianotti, "Intestinal fermentation in vitro models to study food-induced gut microbiota shift: an updated review," *FEMS MICROBIOLOGY LETTERS*, vol. 367, 2020.

[16] J. S. Moon, L. Li, J. Bang, and N. S. Han, "Application of in vitro gut fermentation models to food components: A review," *Food Science and Biotechnology*, vol. 25, pp. 1-7, 2016.

[17] H. Ji, J. Hu, S. Zuo, S. Zhang, M. Li, and S. Nie, "In vitro gastrointestinal digestion and fermentation models and their applications in food carbohydrates," *Crit Rev Food Sci Nutr*, pp. 1-23, 2021.

[18] J. Nsor-Atindana, M. S. Chen, L. Wei, K. M. Noe, Y. Li, and F. Zhong, "Implications of static in vitro digestion of starch in the presence of dietary fiber," *FRONTIERS OF AGRICULTURAL SCIENCE AND ENGINEERING*, vol. 5, pp. 340-350, 2018.

[19] I. A. Brownlee, S. Gill, M. D. Wilcox, J. P. Pearson, and P. I. Chater, "Starch digestion in the upper gastrointestinal tract of humans," *STARCH-STARKE*, vol. 70, 2018.

[20] G. T. Nguyen and P. A. Sopade, "Modeling Starch Digestograms: Computational Characteristics of Kinetic Models for in vitro Starch Digestion in Food Research," *COMPREHENSIVE REVIEWS IN FOOD SCIENCE AND FOOD SAFETY*, vol. 17, pp. 1422-1445, 2018.

[21] S. Marze, "Bioavailability of Nutrients and Micronutrients: Advances in Modeling and In Vitro Approaches," in *ANNUAL REVIEW OF FOOD SCIENCE AND TECHNOLOGY, VOL 8*, vol. 8, M. P. Doyle and T. R. Klaenhammer, Eds., 2017, pp. 35-55.

[22] J. Nadia, J. Bronlund, R. P. Singh, H. Singh, and G. M. Bornhorst, "Structural breakdown of starch-based foods during gastric digestion and its link to glycemic response: In vivo and in vitro considerations," *Comprehensive Reviews in Food Science and Food Safety*, 2021.

[23] G. M. Bornhorst and R. P. Singh, "Bolus Formation and Disintegration during Digestion of Food Carbohydrates," *COMPREHENSIVE REVIEWS IN FOOD SCIENCE AND FOOD SAFETY*, vol. 11, pp. 101-118, 2012.

[24] G. M. Bornhorst, "Gastric Mixing During Food Digestion: Mechanisms and Applications," *Annual Review of Food Science and Technology*, vol. 8, pp. 523-542, 2017.

[25] F. Kong and R. P. Singh, "Disintegration of solid foods in human stomach," *JOURNAL OF FOOD SCIENCE*, vol. 73, pp. R67-R80, 2008.

[26] G. M. Bornhorst and R. P. Singh, "Gastric Digestion In Vivo and In Vitro: How the Structural Aspects of Food Influence the Digestion Process," in *ANNUAL REVIEW OF FOOD SCIENCE AND TECHNOLOGY, VOL 5*, vol. 5, M. P. Doyle and T. R. Klaenhammer, Eds., 2014, pp. 111-132.

[27] G. M. Bornhorst, O. Gouseti, M. S. Wickham, and S. Bakalis, "Engineering Digestion: Multiscale Processes of Food Digestion," *Journal of Food Science*, vol. 81, pp. R534-43, 2016.

[28] R. Lucas-Gonzalez, M. Viuda-Martos, J. A. Perez-Alvarez, and J. Fernandez-Lopez, "In vitro digestion models suitable for foods: Opportunities for new fields of application and challenges," *FOOD RESEARCH INTERNATIONAL*, vol. 107, pp. 423-436, 2018.

[29] S. J. Hur, B. O. Lim, E. A. Decker, and D. J. McClements, "In vitro human digestion models for food applications," *Food Chemistry*, vol. 125, pp. 1-12, 2011.

[30] M. Alminger, A. M. Aura, T. Bohn, C. Dufour, S. N. El, A. Gomes, S. Karakaya, M. C. Martinez-Cuesta, G. J. McDougall, T. Requena, and C. N. Santos, "In Vitro Models for Studying Secondary Plant Metabolite Digestion and Bioaccessibility," *COMPREHENSIVE REVIEWS IN FOOD SCIENCE AND FOOD SAFETY*, vol. 13, pp. 413-436, 2014.

[31] D. Jeong, J. A. Han, Q. Liu, and H. J. Chung, "Effect of processing, storage, and modification on in vitro starch digestion characteristics of food legumes: A review," *FOOD HYDROCOLLOIDS*, vol. 90, pp. 367-376, 2019.

[32] C. Giromini, F. Cheli, R. Rebucci, and A. Baldi, "Invited review: Dairy proteins and bioactive peptides: Modeling digestion and the intestinal barrier," *JOURNAL OF DAIRY SCIENCE*, vol. 102, pp. 929-942, 2019.

[33] D. Kamstrup, R. Berthelsen, P. J. Sassene, A. Selen, and A. Mullertz, "In Vitro Model Simulating Gastro- Intestinal Digestion in the Pediatric Population (Neonates and Young Infants)," *AAPS PHARMSCITECH*, vol. 18, pp. 317-329, 2017.

[34] F. P. Flores and F. Kong, "In Vitro Release Kinetics of Microencapsulated Materials and the Effect of the Food Matrix," in *Annual Review of Food Science and Technology* vol. 8, M. P. Doyle and T. R. Klaenhammer, Eds., 2017, pp. 237-259.

[35] A. Acevedo-Fani and H. Singh, "Biopolymer interactions during gastric digestion: Implications for nutrient delivery," *FOOD HYDROCOLLOIDS*, vol. 116, 2021.

[36] S. Millet, M. J. Van Oeckel, M. Aluwe, E. Delezie, and D. L. De Brabander, "Prediction of In Vivo Short-Chain Fatty Acid Production in Hindgut Fermenting Mammals: Problems and Pitfalls," *CRITICAL REVIEWS IN FOOD SCIENCE AND NUTRITION*, vol. 50, pp. 605-619, 2010.

[37] Z. Amanzougarene and M. Fondevila, "Fitting of the In Vitro Gas Production Technique to the Study of High Concentrate Diets," *ANIMALS*, vol. 10, 2020.

[38] S. Tassone, R. Fortina, and P. G. Peiretti, "In Vitro Techniques Using the Daisy(II) Incubator for the Assessment of Digestibility: A Review," *Animals (Basel)*, vol. 10, 2020.

[39] F. J. Moyano, M. A. Saénz de Rodrigáñez, M. Díaz, and A. G. J. Tacon, "Application of in vitro digestibility methods in aquaculture: constraints and perspectives," *Reviews in Aquaculture*, vol. 7, pp. 223-242, 2015.

**References for the coded number in Table 3 and Table 4.**

[1] G. Bitterlich, "Digestive processes in silver carp (Hypophthalmichthys molitrix) studied in vitro," *Aquaculture*, vol. 50, pp. 123-131, 1985.

[2] A. E. Eid and A. J. Matty, "A simple in vitro method for measuring protein digestibility," *Aquaculture*, vol. 79, pp. 111-119, 1989.

[3] C. Sturmbauer, "Different enzymes for laminarine digestion in Chondrostoma nasus (cyprinidae) and Oreochromis sp. (cichlidae)," *Comparative Biochemistry and Physiology Part A: Physiology*, vol. 100, pp. 199-202, 1991.

[4] W. M. Koven, J. R. Henderson, and J. R. Sargent, "Lipid digestion in turbot (Scophthalmus maximus) in-vivo and in-vitro studies of lipolytic acitivity in various segments of the digestive tract," *Aquaculture*, vol. 151, pp. 155 - 171, 1997.

[5] E. F. Gomes, A. O. Teles, A. Gouveia, and P. Rema, "In vivo and in vitro digestibility of diets and feedstuffs for rainbow trout (Oncorhynchus mykiss)," *Journal of Applied Ichthyology*, vol. 14, pp. 109-111, 1998.

[6] M. Bassompierre, H. R. Kristiansen, and E. McLean, "Influence of weight upon in vitro protein digestion in rainbow trout," *Journal of Fish Biology*, vol. 52, pp. 213-216, 1998.

[7] C. G. Carter, M. P. Bransden, R. J. van Barneveld, and S. M. Clarke, "Alternative methods for nutrition research on the southern bluefin tuna, Thunnus maccoyii: in vitro digestibility," *Aquaculture*, vol. 179, pp. 57-70, 1999.

[8] A. García-Ortega, A. Koussoulaki, H. Boer, and J. Verreth, "In vitro protein digestibility of Artemia decapsulated cysts and nauplii, and of microbound diets for larval fish," *Aquaculture Research*, vol. 31, pp. 475-477, 2000.

[9] V. Weerasinghe, R. W. Hardy, and N. F. Haard, "An in vitro method to determine phosphorus digestibility of rainbow trout Oncorhynchus mykiss (Walbaum) feed ingredients," *Aquaculture Nutrition*, vol. 7, pp. 1-9, 2001.

[10] K. Rungruangsak-Torrissen, A. Rustad, J. Sunde, S. A. Eiane, H. B. Jensen, J. Opstvedt, E. Nygård, T. A. Samuelsen, H. Mundheim, U. Luzzana, and G. Venturini, "In vitro digestibility based on fish crude enzyme extract for prediction of feed quality in growth trials," *Journal of the Science of Food and Agriculture*, vol. 82, pp. 644-654, 2002.

[11] S. K. Tonheim, A. Nordgreen, I. Høgøy, K. Hamre, and I. Rønnestad, "In vitro digestibility of water-soluble and water-insoluble protein fractions of some common fish larval feeds and feed ingredients," *Aquaculture*, vol. 262, pp. 426-435, 2007.

[12] A. Nordgreen, S. Tonheim, and K. Hamre, "Protein quality of larval feed with increased concentration of hydrolysed protein: effects of heat treatment and leaching," *Aquaculture Nutrition*, vol. 15, pp. 525-536, 2009.

[13] M. Á. Sáenz de Rodrigáñez, E. Medina, F. J. Moyano, and F. J. Alarcón, "Evaluation of protein hydrolysis in raw sources by digestive proteases of Senegalese sole (Solea senegalensis, Kaup 1858) using a combination of an in vitro assay and sodium dodecyl sulphate polyacrylamide gel electrophoresis analysis of products," *Aquaculture Research*, vol. 42, pp. 1639-1652, 2011.

[14] C. Aguilera, R. Mendoza, I. Iracheta, and G. Marquez, "Digestive enzymatic activity on Tropical gar (Atractosteus tropicus) larvae fed different diets," *Fish Physiology and Biochemistry*, vol. 38, pp. 679-691, 2012.

[15] K. Thongprajukaew, U. Kovitvadhi, S. Kovitvadhi, A. Engkagul, and K. Rungruangsak-Torrissen, "Evaluation of growth performance and nutritional quality of diets using digestive enzyme markers and in vitro digestibility in Siamese fighting fish (Betta splendens Regan, 1910)," *African Journal of Biotechnology*, vol. 12, 2013.

[16] S. Fenerci and E. Şener, "In vivo and in vitro protein digestibility of rainbow trout (Oncorhynchus mykiss Walbaum, 1972) fed steam pressured or extruded feeds," *Turkish Journal of Fisheries and Aquatic Sciences*, vol. 5, 2005.

[17] H. Ali, M. M. Haque, M. M. R. Chowdhury, and M. I. Shariful, "In vitro protein digestibility of different feed ingredients in Thai koi (Anabas testudineus)," *Journal of the Bangladesh Agricultural University*, vol. 7, pp. 205-210, 2009.

[18] Z. Sultana, S. Ahmed, S. Iqball, and A. H. Chisty, "Determination of in vitro protein digestibility of different feed ingredients for Nilotica (Oreochromis nilotica)," *Bangladesh Research Publication Journal*, vol. 4, pp. 87-94, 2010.

[19] J. S. Anderson, S. P. Lall, D. M. Anderson, and M. A. McNiven, "Evaluation of protein quality in fish meal by chemical and biological assays," *Aquaculture*, vol. 115, pp. 305 - 325, 1993.

[20] F. M. Dong, R. W. Hardy, N. F. Haard, F. T. Barrows, B. A. Rasco, W. T. Fairgrieve, and I. P. Forster, "Chemical composition and protein digestibility of poultry by-product meals for salmonid diets," *Aquaculture*, vol. 116, pp. 149-158, 1993.

[21] M. Grabner, "An in vitro method for measuring protein digestibility of fish feed components," *Aquaculture*, vol. 48, pp. 97 - 110, 1985.

[22] L. E. Dimes and N. F. Haard, "Estimation of protein digestibility - I. Development of an in vitro method for estimating protein digestibility in salmonids (Salmon gairdneri)," *Comparative Biochemistry and Physiology*, vol. 108, pp. 249 - 362, 1994.

[23] L. E. Dimes, F. L. Garcia-Carreno, and N. F. Haard, "Estimation of protein digestibility—III. Studies on the digestive enzymes from the pyloric ceca of rainbow trout and salmon," *Comparative Biochemistry and Physiology Part A: Physiology*, vol. 109, pp. 349-360, 1994.

[24] N. F. Haard, L. E. Dimes, R. E. Arndt, and F. M. Dong, "Estimation of Protein Digestibility—IV. Digestive Proteinases from the Pyloric Caeca of Coho Salmon (<i>Oncorhynchus kisutch</i>) Fed Diets Containing Soybean Meal," *Comparative Biochemistry and Physiology Part B: Biochemistry and Molecular Biology*, vol. 115, pp. 533-540, 1996.

[25] J. S. Anderson, D. A. Higgs, R. M. Beames, and M. Rowshandeli, "Fish meal quality assessment for Atlantic salmon (Salmo salar L.) reared in sea water," *Aquaculture Nutrition*, vol. 3, pp. 25-38, 1997.

[26] F. J. Alarcon, F. J. Moyano, M. Diaz, C. Fernandez-diaz, and M. Yufera, "Optimization of the protein fraction of microcapsules used in feeding of marine fish larvae using in vitro digestibility techniques," *Aquaculture Nutrition*, vol. 5, pp. 107-113, 1999.

[27] S. M. Tibbetts, J. A. J. Verreth, and S. P. Lall, "In vitro pH-Stat protein hydrolysis of feed ingredients for Atlantic cod, Gadus morhua. 2. In vitro protein digestibility of common and alternative feed ingredients," *Aquaculture*, vol. 319, pp. 407-416, 2011.

[28] E. Martínez-Montaño and J. P. Lazo, "In Vitro Protein Digestibility of Dietary Ingredients Throughout Ontogeny of California Halibut, Paralichthys californicus, Larvae," *Journal of the World Aquaculture Society*, vol. 43, pp. 51-62, 2012.

[29] C. Minjarez-Osorio, M. L. González-Félix, and M. Perez-Velazquez, "Biological performance of Totoaba macdonaldi in response to dietary protein level," *Aquaculture*, vol. 362-363, pp. 50-54, 2012.

[30] M. Bassompierre, T. Børresen, P. Sandfeld, B. Rønsholdt, W. Zimmermann, and E. McLean, "An evaluation of open and closed systems for in vitro protein digestion of fish meal," *Aquaculture Nutrition*, vol. 3, pp. 153-159, 1997.

[31] M. A. SÁEnz de RodrigÁÑEz, B. Gander, M. Alaiz, and F. J. Moyano, "Physico-chemical characterization and in vitro digestibility of commercial feeds used in weaning of marine fish," *Aquaculture Nutrition*, vol. 17, pp. 429-440, 2011.

[32] M. Grabner and R. Hofer, "The digestibility of the Proteins of Broad Bean (Vicia Faba) and Soya Bean (Glycine Max) Under In-vitro conditions Simulating the Alimentary Tracts of Rainbow Trout (Salmo gairdneri) and Carp (Cyprinus carpio)," *Aquaculture*, vol. 48, pp. 111-122, 1985.

[33] M. Bassompierre, A. Kjrer, and E. McLean, "Simulating protein digestion on trout a rapid and inexpensive method for documenting fish meal quality and screening novel protein sources for use in aquafeeds," *Croatian Journal of Fisheries: Ribarstvo*, vol. 55, pp. 137-145, 1997.

[34] F. J. Alarcón, F. J. Moyano, and M. Díaz, "Evaluation of different protein sources for aquafeeds by an optimised pH-stat system," *Journal of the Science of Food and Agriculture*, vol. 82, pp. 697-704, 2002.

[35] E. Martínez-Montaño, E. Peña, U. Focken, and M. T. Viana, "Intestinal absorption of amino acids in the Pacific bluefin tuna (Thunnus orientalis): In vitro uptake of amino acids using hydrolyzed sardine muscle at three different concentrations," *Aquaculture*, vol. 299, pp. 134-139, 2010.

[36] F. J. Moyano and S. Laurent, "Comparison of in vitro systems of protein digestion using either mammal or fish proteolytic enzymes," *Comparative Biochemistry and Physiology*, vol. 128, pp. 359-368, 2001.

[37] M. Hamdan, F. J. Moyano, and D. Schuhardt, "Optimization of a gastrointestinal model applicable to the evaluation of bioaccessibility in fish feeds," *Journal of the Science of Food and Agriculture*, vol. 89, pp. 1195-1201, 2009.

[38] G. A. Morales and F. J. Moyano, "Application of an in vitro gastrointestinal model to evaluate nitrogen and phosphorus bioaccessibility and bioavailability in fish feed ingredients," *Aquaculture*, vol. 306, pp. 244-251, 2010.

[39] T. Morken, F. J. Moyano, L. Márquez, M. Sørensen, L. T. Mydland, and M. Øverland, "Effects of autoclaving and sodium diformate supplementation to diets on amino acid composition, in vivo digestibility in mink (Neovison vison) and in vitro bioavailability using digestive enzymes from Atlantic salmon (Salmo salar)," *Animal Feed Science and Technology*, vol. 178, pp. 84-94, 2012.

[40] L. Márquez, M. Øverland, S. Martínez-Llorens, T. Morken, and F. J. Moyano, "Use of a gastrointestinal model to assess potential amino acid bioavailability in diets for rainbow trout (Oncorrhynchus mykiss)," *Aquaculture*, vol. 384-387, pp. 46-55, 2013.

[41] F. Yasumaru and D. Lemos, "Species specific in vitro protein digestion (pH-stat) for fish: method development and application for juvenile rainbow trout (Oncorhynchus mykiss), cobia (Rachycentron canadum), and Nile tilapia (Oreochromis niloticus)," *Aquaculture*, vol. 426-427, pp. 74-84, 2014.

[42] A. I. Román-Gavilanes, E. Martínez-Montaño, and M. T. Viana, "Comparative Characterization of Enzymatic Digestion from Fish and Soybean Meal from Simulated Digestive Process of Pacific Bluefin Tuna, Thunnus orientalis," *Journal of the World Aquaculture Society*, vol. 46, pp. 409-420, 2015.

[43] E. Castillo-Lopez, R. E. Espinoza-Villegas, and M. T. Viana, "In vitro digestion comparison from fish and poultry by-product meals from simulated digestive process at different times of the Pacific Bluefin tuna, Thunnus orientalis," *Aquaculture*, vol. 458, pp. 187-194, 2016.

[44] S. Rahmah, M. Aliyu-Paiko, and R. Hashim, "In vivo and in vitro protein digestibility in juvenile bagrid catfish Mystus nemurus (Cuvier and Valenciennes 1840) fed soybean meal-based diets," *Aquaculture Research*, vol. 47, pp. 1392-1401, 2016.

[45] E. Peña, C. Hernández, L. Ibarra-Castro, and C. A. Álvarez-González, "In vitro protein digestibility of different grow-out stages of spotted rose snapper (Lutjanus guttatus, Steindachner, 1869)," *Aquaculture Nutrition*, vol. 23, pp. 1204-1215, 2017.

[46] I. Trejo-Escamilla, M. A. Galaviz, M. Flores-Ibarra, C. A. Álvarez González, and L. M. López, "Replacement of fishmeal by soya protein concentrate in the diets ofTotoaba macdonaldi(Gilbert, 1890) juveniles: effect on the growth performance,in vitrodigestibility, digestive enzymes and the haematological and biochemistry parameters," *Aquaculture Research*, vol. 48, pp. 4038-4057, 2017.

[47] S. M. Tibbetts, F. Yasumaru, and D. Lemos, "In vitro prediction of digestible protein content of marine microalgae (Nannochloropsis granulata) meals for Pacific white shrimp (Litopenaeus vannamei) and rainbow trout (Oncorhynchus mykiss)," *Algal Research*, vol. 21, pp. 76-80, 2017.

[48] N. Gilannejad, G. Martinez-Rodriguez, M. Yufera, and F. J. Moyano, "Modelling digestive hydrolysis of nutrients in fish using factorial designs and desirability function," *PLoS One*, vol. 13, pp. e0206556, 2018.

[49] I. Lemus, C. Maldonado, G. Cuzon, A. Sanchez, G. Gaxiola, A. Alvarez, and M. Guerrero, "In Vitro and In Vivo Feedstuff Digestibility for Snook, Centropomus undecimalis, Juveniles," *Journal of the World Aquaculture Society*, vol. 49, pp. 205-215, 2018.

[50] M. K. Mirzakhani, A. Abedian Kenari, and A. Motamedzadegan, "Prediction of apparent protein digestibility by in vitro pH-stat degree of protein hydrolysis with species-specific enzymes for Siberian sturgeon (Acipenser baeri, Brandt 1869)," *Aquaculture*, vol. 496, pp. 73-78, 2018.

[51] M. J. Lewis, D. S. Francis, D. Blyth, F. J. Moyano, R. P. Smullen, G. M. Turchini, and M. A. Booth, "A comparison of in-vivo and in-vitro methods for assessing the digestibility of poultry by-product meals using barramundi (lates calcarifer); impacts of cooking temperature and raw material freshness," *Aquaculture*, vol. 498, pp. 187-200, 2019.

[52] A. J. Vizcaíno, M. I. Sáez, T. F. Martínez, F. G. Acién, and F. J. Alarcón, "Differential hydrolysis of proteins of four microalgae by the digestive enzymes of gilthead sea bream and Senegalese sole," *Algal Research*, vol. 37, pp. 145-153, 2019.

[53] R. E. Cian, C. Bacchetta, J. Cazenave, and S. R. Drago, "In vitro assays predicts mineral retention and apparent protein digestibility of different fish feed measured using a juvenileP. mesopotamicusmodel," *Aquaculture Research*, vol. 49, pp. 2267-2277, 2018.

[54] F. J. Toledo‐Solís, R. Martínez‐García, M. Díaz, E. S. Peña Marín, M. P. Di Yorio, P. G. Vissio, C. A. Álvarez‐González, and M. Saenz de Rodrigáñez, "Potential bioavailability of protein and lipids in feed ingredients for the three‐spot cichlid Amphilophus trimaculatus : An in vitro assessment," *Aquaculture Research*, vol. 51, pp. 2913-2925, 2020.

[55] M. S. Silva, P. A. J. Prabhu, R. Ornsrud, V. Sele, S. Krockel, J. J. Sloth, and H. Amlund, "In vitro digestion method to evaluate solubility of dietary zinc, selenium and manganese in salmonid diets," *J Trace Elem Med Biol*, vol. 57, pp. 126418, 2020.

[56] X. D. Duan, L. Feng, W. D. Jiang, P. Wu, Y. Liu, J. Jiang, B. P. Tan, Q. H. Yang, S. Y. Kuang, L. Tang, and X. Q. Zhou, "The dynamic process of dietary soybean beta-conglycinin in digestion, absorption, and metabolism among different intestinal segments in grass carp (Ctenopharyngodon idella)," *Fish Physiol Biochem*, vol. 46, pp. 1361-1374, 2020.

[57] S. Rahmah, R. Hashim, and A. F. M. El‐Sayed, "Digestive proteases and in vitro protein digestibility in bagrid catfish Mystus nemurus (Cuvier and Valenciennes 1840)," *Aquaculture Research*, vol. 51, pp. 4613-4622, 2020.
